# Ultraviolet Radiation Albedo and Reflectance in Review: The Influence to Ultraviolet Exposure in Occupational Settings

**DOI:** 10.3390/ijerph15071507

**Published:** 2018-07-17

**Authors:** Joanna Turner, Alfio V. Parisi

**Affiliations:** Faculty of Health Engineering and Sciences, University of Southern Queensland, Toowoomba 4350, Australia; alfio.parisi@usq.edu.au

**Keywords:** UV radiation, albedo, reflectance, UV exposure

## Abstract

Ultraviolet (UV) albedo and UV reflectance are defined, compared and contrasted, to explain their roles and place in studies focusing on UV radiation and exposure measurements, in the context of localised albedo measurement and human UV exposure studies. This review recommends that the term UV albedo be used when investigating natural horizontal surfaces when the albedo is not known to change significantly over time. The term UV reflectance should be mostly used for non-natural surfaces and non-horizontal measurements and will change with respect to the geometry of the irradiances reflected and received, and due to the intrinsic nature of the surface itself. UV albedo measurements made in the literature have been compiled, in both broadband and spectral UV albedo measurements. Broadband measurements have been tabulated and spectral UV measurements have been displayed visually. The methodology of measurements is briefly discussed. Finally, studies that consider how high albedo or reflectance sites influence UV exposure are reviewed. It was concluded that there is currently no known relationship between the albedo or reflectance of a surface and the resulting influence it has on individual UV exposure. This presents an opportunity for researchers to continue exploring the influence of reflective UV surfaces.

## 1. Introduction

Ultraviolet (UV) radiation is both advantageous and disadvantageous to a variety of species in the biosphere, depending on levels of UV exposure [1]. In humans, the contrasting issues of advantage versus disadvantage has to be managed by balancing UV exposure, because failure to do so can contribute to increased risks of opposing health effects [2,3]. Too little exposure contributes to reduced vitamin D production, which in turn contributes to poor calcium absorption, decreased bone health and a variety of other potential health concerns [4]. In turn, too much exposure contributes to increased risk in development of skin cancer, and the direct relationship between exposure and cancer induction is an ongoing investigation for health researchers [5]. Health effects are not limited to these two opposing issues as flora and fauna are additionally affected in the biosphere, but for the purposes of this review, the influence on health effects in humans will be used as the main focus of our knowledge of UV radiation.

There are many studies which have reviewed effects of personal UV radiation exposure, which range from purposeful (such as sun bathing) to non-purposeful exposure (which can include occupational and non-occupational). There are a range of factors that influence the day-to-day and site specific UV exposure to humans, all of which have been investigated in some capacity, including solar elevation angle (time of day), weather factors including clouds, time of year, total ozone column, aerosol index, diffuse or direct exposure due to scattering and absorption through the atmosphere, and scattering due to immediate surroundings [6]. Shading is a significantly investigated topic, with public understanding of UV exposure under shade often misaligning with current research [7]. The final factor that contributes to influence UV exposure is reflectance from surfaces, which is sometimes referred to as albedo. This review will focus on UV reflectance, which until now, has not been collectively reviewed in regards to local or nearby surfaces that influence UV exposure.

Influence of albedo or reflectance on UV exposure is not an expansive research area compared to other UV influencing factors. At least, it has not been prioritised in many studies, presumably due to the reason that some surfaces may not be highly reflective within the UV spectrum, and have low influence on UV exposure. However, there are enough surfaces that do reflect UV radiation effectively, that a collective review of the current state of research would provide a platform to determine if more information is collectively required.

A related area that appears to be lacking within knowledge surrounding UV exposure, is exactly how much albedo or reflectance from a surface contributes to changes in UV exposure. In particular, there is only a limited range of studies that have measured empirical UV albedo or reflectance measurements. This means that collectively there is uncertainty on agreement between UV albedo or reflectance measurements across the current published research, and if their values contribute consistently to knowledge around UV exposure influence. In order to address the larger question of influence on UV exposure, there is a need to review the literature available on UV albedo and reflectance measurements to determine consistency in measurement. It has been informally observed that in cases where UV albedo or reflectance is required for a project, many studies will measure this individually, but often do not report the value, or the method. Some studies assume research from other literature will be appropriate despite variation in location or surface composition, choosing the most appropriate values available, without confirming that these are the most appropriate reflectance values. It is hoped that for the future, confidence in albedo or reflectance measurements will be supported by this review, and provide some recommendations for the method of measurement, should further data be required in a study. The review will present the following information:

- Definitions of albedo and reflectance, including the range of how the terms are used in the literature.
- Reported albedo and reflectance measurements focusing on local or immediate surroundings with specific focus on UV albedo or reflectance. Total solar albedo or reflectance papers that do not specifically refer to UV radiation will not be considered here.
- Similar surface types will be grouped for comparison and visually analysed if sufficient data is available, otherwise data will be tabulated.
- Broadband and spectral measurements will be presented separately.
- Method of measurement, including reviewing range of devices implemented.
- Review the influence of UV albedo/reflectance on individual UV exposure.

Limitations imposed on the review also include restricting albedo or reflectance measurements to mostly empirical studies. Reference will be made to modelled studies when relevant and look at localised measurements rather than satellite measured albedo. This will not preclude discussing any conclusions from such studies but seeks to restrict reflectance measurements to a similar method of data collection. From this restriction the authors anticipate looking at situations where humans are in close vicinity to the reflective surface, thus accommodating the question of influence over local UV exposure.

## 2. Albedo and Reflectance Definitions

Surprisingly the definitions of these physical processes vary, indeed Kukla [8] laments that of the term albedo “Usages of the term vary and definitions are rare.” Despite the above comment regarding restricting review of studies to localised measurements, the literature within remote sensing publications are useful to define and streamline the use of the terms albedo and reflectance. The authors are not suggesting that either term’s definition is confusing, but instead are suggesting that recognising when to use each term and what it implies is crucial for localised measurements, specifically in regard to surface orientation and irradiance geometry. These definitions can prove useful when contrasting against broad scale measurements made in remote sensing applications. Specifically, the data collection method, and physical behaviour of electromagnetic radiation is the key to ensuring consistency amongst albedo and reflectance reporting. This is particularly relevant when reporting non-horizontal surface measurements.

Coakley [9] provides a number of relevant factors to understand albedo. The word albedo, meaning “whiteness”, is derived from Latin, and conjures the idea of haziness, but is also useful to imagine visually. Albedo is defined as the fraction of incident sunlight that the surface reflects. The standard use of the definition of albedo in ultraviolet radiation studies (and the remaining solar spectrum) is the ratio of reflected (upwelling) irradiance to incident (downwelling) irradiance. For broadband measurements this is simply:

(1) $$a=\frac{I_{u}}{I_{d}}$$

where a is the albedo, $I_{u}$ is the total reflected or upwelling irradiance, and $I_{d}$ is the total downwelling or incident irradiance from the surface measured across the wavelength spectrum. For spectral albedo measurements, this same calculation can be performed per wavelength increment across the desired spectrum.

When it comes to ensuring the definition of albedo is determined correctly, it is important to consider the specific terms used alongside albedo and their associated assumptions. For remote sensing, albedo is more commonly referred to as surface albedo. The first and most important approximation [9] is that a surface reflects isotropically, indicating that reflected intensity is independent of incident irradiance angles, thus implying that reflected irradiance is constant in intensity and distributed in all possible directions. This type of surface is also known as a Lambertian reflector or surface [10]. The name is derived from Lambert’s Law, which is essentially the definition of isotropic, in that Lambert’s Law (derived empirically) states the brightness of a surface is independent of the angle in which it is viewed [11]. A perfect Lambertian surface is therefore a perfect diffuse surface, and while this does not exist in nature, snow is the most likely surface to approach this definition [9]. These sources of literature focus on remote sensing or planetary albedo, which includes the entire solar spectrum reaching the Earth’s atmosphere, or alternatively the radiation penetrating the atmosphere to the Earth’s surface.

Hapke [11] defines reflectance or reflectivity as the fraction of incident light scattered or reflected by a material. Hapke also states that reflectance has the connotation of diffuse reflection, whilst reflectivity suggested specular reflection occurring physically. At first glance then, reflectance would appear to be equivalent to albedo making the assumption of a Lambertian surface. However, Hapke continues to sub-define types of reflectance, and we can use these definitions to guide our current understanding. Reflectance can be preceded by certain descriptors that describe the range of collimation of the radiation source and the radiation detected (Table 1). When used to describe both the radiation emitted by the source and radiation captured at the detector, an accurate description of the physical behaviour of radiation is obtained. Hapke [11] provides the example of directional-hemispherical reflectance. The former descriptor indicates the source is highly collimated, whilst the latter description indicates the reflected and scattered radiation is distributed in all directions. Hapke also provides some other basic examples. The sun sub-tends approximately 0.5° of the sky, thus can be treated as having a range of collimation depending on atmospheric conditions. A sunny day may provide near directional radiation from the source, whereas on a cloudy day, collimation may become hemispherical. As a result, depending on the surface being irradiated, measurements may range from bidirectional reflectance, to bihemispherical reflectance. Coakley [9] reminds us that an albedo is not an instrinsic property of a surface, and we can see from Hapke’s definitions that the spectral and angular range of radiation strongly controls the derived values of reflectance. Why then would we want to separate albedo and reflectance and their definitions? This comes back to the surface itself. Coakley says that albedo is not an intrinsic property of a surface, therefore we cannot use albedo to describe the physical behaviour of radiation at the surface boundary when we know that the surface itself is affecting the physical behaviour of radiation reflected. This brings us back to determining if the reflection itself is truly diffuse, or some other reflective behaviour, such as specular, or some combination of specular and diffuse reflectance, is occurring.

Bacon [12] defines surface reflection “as the abrupt change in the electrical properties of the medium through which electromagnetic radiation passes”. Radiant flux density (irradiance) received and reflected at the surface of a material follows Fresnel’s Law [10] which states that the reflected angle of irradiance will be equal to the angle of irradiance incidence. This definition assumes that the surface is flat and smooth, at least with respect to the incident radiation wavelength. Fresnel reflection produces specular reflectance. The Rayleigh roughness criterion can then be used to determine whether reflection or scattering will take place.

The production of specular reflectance is therefore part of the intrinsic property of the surface, which means that change in intensity of reflectance from a surface is due to variation between a perfectly flat and perfectly rough surface (which most surfaces are) and is related directly to particle, molecule or polymer interface interaction with incident irradiance. Ahn et al. [13] has demonstrated that the proportion of specular reflectance to diffuse reflectance is relative to particle size, with specular reflectance increasing with decreasing particle size, and diffuse reflectance increasing with increasing particle size on nickel coated particle polyelectrolyte monolayers. Not all reflection occurs at the surface boundary, with penetration of radiation into the surface layer possible, as well as being reflected at any possible angle and direction, which corresponds to a diffuse reflector. How then does this knowledge help us to address definitions of albedo and reflectance within UV research?

A review of the literature involving measurement of UV albedo, shows that the majority of studies use the definition provided at the beginning of this section: the ratio of upwelling radiation to downwelling radiation. This definition in many studies appears to imply hemispherical downwelling irradiance and either conical or hemispherical upwelling irradiance. Sliney [14] provides examples of the types of sensor measurements used in environmental UV studies and the irradiance detection types including hemispherical and conical definitions according to the cosine response. Many UV albedo studies also focus on natural surfaces, which tend to be more diffuse reflectors with more surface roughness (see Table 2). Finally, many of these studies use horizontal surfaces because many natural surfaces are approximately horizontal and for remote sensing purposes may be considered an approximate flat surface. As previously described, Hapke [15] describes this as bihemispherical reflectance, but also indicates this is equivalent to Bond albedo or spherical albedo of a planet. The range of terminology available to describe geometry of reflectance is therefore varied, but on the whole useful for equating reflectance measured to some overall expression of information.

Therefore, for horizontal natural surfaces, albedo is the most appropriate measurement defined here, and accommodates the associated assumptions satisfactorily in most cases. When we consider non-horizontal surfaces, work by Wiehs [16] shows that the albedo does not remain constant. This is due to surrounding topography and the ratio of direct to diffuse irradiance, which as a result suggests changes in directional reflectivity. Weihs concludes after a review of the relevant literature for non-horizontal surfaces, that most ground surfaces actually reflect some of the incoming irradiance specularly. This changes directional reflectivity for a surface, and for a nearby irradiance sensor. This, combined with anisotropic UV sky irradiance, means that maps of varying directional reflectivity (described as albedo in the study) can be created for natural surfaces of varying surface orientation. This work was solely focused in a snow covered terrain and produced topographical maps of albedo variation, with values of similar albedo presented similarly to a meteorological map. It is therefore difficult to work out if in this example, differentiating between albedo and reflectance is useful given the range of ways the terms are used across research. Therefore, it is probably more useful to determine when albedo (and its associated assumptions and conditions) is not occurring.

Improved visualisation of the process of surface reflectance can be provided by computer modelling visualisation processes such as demonstrated by Nayar et al. [17] using polar plots. The plots describe the specular spike, specular lobe and diffuse lobe, which can be used as primary building blocks to build a way to model a surface to show variable reflectance. Being able to visualise what albedo and reflectance could look like if we could physically observe the reflectance of UV radiation, may be helpful. For example, we can use this to ask, when might specular changes be observed? This can be answered by returning to the assumed conditions surrounding albedo.

The assumed conditions of albedo are not satisfied when we start looking at man-made flat surfaces (such as metals), with or without non-horizontal surfaces. Given their normal geometric construction, man-made surfaces will receive differing incident irradiance values compared to a horizontal surface due to the cosine response [18,19]. This means that depending on the position of the sun in the sky, the surface will reflect the proportional incident intensity relative to the cosine response adjusted irradiance. Thus, albedo measurements (which rely on downwelling rather than Sun normal measurements) from these surfaces in these conditions become artificially inflated and no longer stay within the 0 to 1 (or 0–100%) scale of albedo. This was demonstrated by Turner and Parisi [20] with reflectance measured from differing orientations of construction materials. In that study, the conclusion was to use the term reflection to differentiate between measurements adhering to albedo definitions and assumptions (horizontal surface) and to reflectance definitions (non-horizontal surfaces in order to compare against horizontal measurements). In a more recent paper, it was concluded that reflectance was a more appropriate term, and would continue to be, particularly given the above discussion presented here [21]. Therefore, we can conclude that unless the conditions and assumptions for albedo are satisfied (horizontal surface and assumed Lambertian reflector), it is better to use reflectance to describe the radiative transfers occurring at a surface.

## 3. Albedo and Reflectance Measurements

The literature focused on in this review will be on local or immediate albedo or reflectance, rather than satellite measured or calculated albedo. The main reason for this focus is that satellite derived albedo may account for more than one surface type, whereas localised albedo will be focused on one specific surface type per measurement (where a short distance between the surface and sensor will encapsulate a single surface). The majority of literature surveyed in this review provides UV albedo measurements, acceding to the albedo measurement (Equation (1)) with the assumptions of isotropic reflected irradiance. In this review of published albedo, we will begin with broadband albedo either measured directly with a broadband instrument, or calculated from spectral measurements. Albedo measurements made only at specific individual wavelengths have not been included in this section. In this review, albedo (or reflectance) will be reported as a range of 0 to 100%.

### 3.1. UV Albedo Measurements—Broadband

Table 2 provides a list of published broadband albedo or reflectance measurements [14,22,23,24,25,26,27,28,29,30,31,32,33,34,35,36], based upon measurements made with the sun as the main source of irradiance, except in one or two instances where an artificial UV source has been used. The identification number of each publication in Table 2 is used in Table 3 and Table 4. This number is not the same as the reference number. Table 3 summarises all known published broadband UV albedo measurements for a variety of natural surfaces, while Table 4 summarises broadband UV albedo measurements for man-made surfaces. It is assumed in all these measurements that they have been conducted on horizontal surfaces, given the methodology reported in each documented study. Table 4 also includes data from two studies [31,33] but Table 3 does not as they do not include any natural surface data. Later on, in this review Berdahl and Bretz, and Parker et al. [22,33] are compared as they each review reflectance from commercial products, of which there is an overlap of similar surface types, which is presented in Table 5 for comparison.

Table 3 shows that surprisingly there are very few repeatedly reported albedo measurements. It appears that most surfaces have a limited number of reported albedo values, except for particularly common surfaces such as grass, sand and snow, of which many are distinguished by different types of each surface with particular descriptions. This gives rise to the issue of being able to compare UV albedo across different studies. Extra description for each surface does not necessarily mean that comparisons can be made easily. Grass albedo measurements show that it is the composition of the surface that is the factor that causes variation in albedo measurement as is shown by [25,26] where Diffey et al., shows variation in grass albedo from different countries, while Correa and Ceballos show the difference in green and yellow grass albedo. These contrast strongly with the assumptions made by Coakley [9] which suggests the assumptions are not appropriate to be incorporated even for localised UV albedo. Equally snow, reported as the surface that could approach a perfect diffuse reflecting surface, shows significant variation in UV albedo. There are many studies that investigate the changing snow albedo but only a few are cited here [37,38,39,40,41].

In the “Protecting Workers from Ultraviolet Radiation” report [29], there are two different sets of reported albedo values, with one contrasting measurement. It shows surf reported in one section as 20%, compared to 25–30% in another. Further review of the document did not provide further information as to why the reported values appear to differ for some surface types, and whether these differences were due to different methodologies of collection of the data. Another paper included in the reported list is by Heisler and Grant [28], as it is regularly cited as reporting albedo measurements, although it would appear that it is actually citing two other papers (listed in Table 2) [14,23]. The decision to include the paper, was simply for consistency with the literature of frequently cited papers.

In Table 4 the most commonly repeated UV albedo measurements are concrete, asphalt and tarmac. Asphalt (using the American English spelling) is also commonly known as bitumen in other English speaking countries. Tarmac could also be equally used interchangeably with asphalt, as it is defined as a material of broken stones mixed with tar. In some countries tarmac is recognised as a trademark. Readers of published albedo papers are therefore required to recognise synonyms of material names for a surface type. The decision to leave all the descriptive terms in Table 4, rather than combining them, are used to illustrate how simple language differences may show how difficult it may be to make comparisons between localised UV albedo of surfaces that are colloquially described or described even by manufacturer name. It may be that authors or conductors of studies are simply uncertain of the exact ingredients contained within a surface type, as that would require them to be cognisant of stone and gravel types used in the tar or pitch mix used to seal a road or ground surface. Concrete and asphalt (tarmac) show a similar range of UV albedo values, ranging from 2 to 15% albedo. Concrete similarly has the issue of different ingredients potentially influencing the UV albedo. A study conducted by Levinson and Akbari [42] looked at the solar albedo (total solar spectrum) rather than UV albedo, however they found composition greatly affected the albedos’ produced, with concrete albedo values strongly influenced by sand albedo, cement albedo and rock (weathering albedo). Despite the study not specifically looking at UV albedo, we can infer from the study, that there may be variation in concrete UV albedo. Weathering of course, can depend on the surface in question also. Kultur et al. [43] found that for metal, cement and bitumen surfaces, weathering (defined as after one year) did not significantly decrease albedo for cement and bitumen compared to non-weathered products. However, this particular study was conducted in the laboratory, and assumed that the surfaces exposed to UV radiation between the wavelengths of 300–380 nm were simulated in aging through degradation of surface type. There was no information regarding the metals after one year. Damage of materials due to UV radiation is an important topic area for materials research, but will not be discussed further here.

### 3.2. UV Albedo Measurements—Spectral

**Figure 1:**
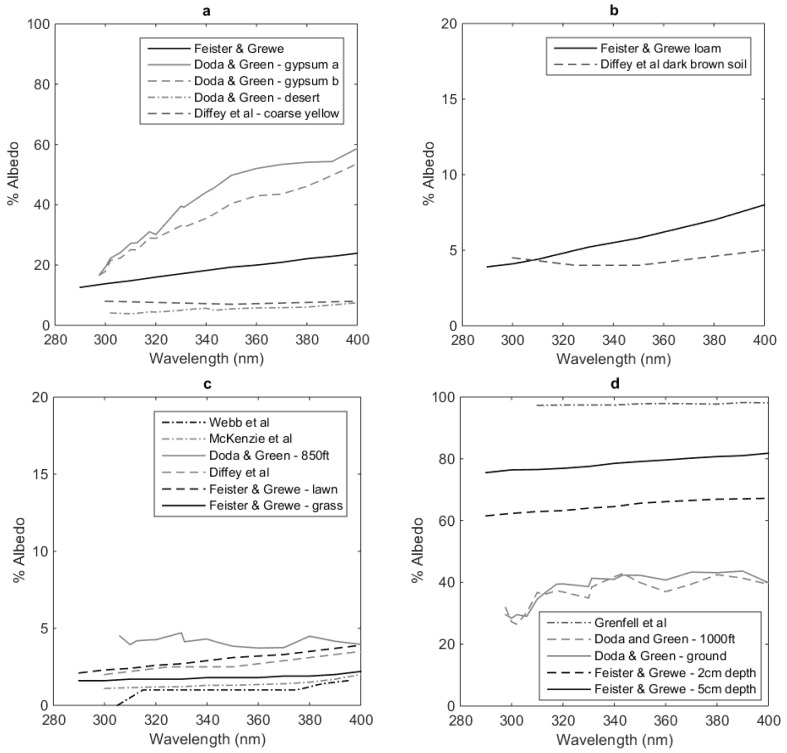
Spectral UV albedo for (**a**) sand, (**b**) earth, (**c**) grass and (**d**) snow. The data has been collated from Feister and Grewe [27], Doda and Green [44,45], Diffey et al. [26], Webb et al. [36], Grenfell et al. [37] and McKenzie et al. [32].

**Figure 2:**
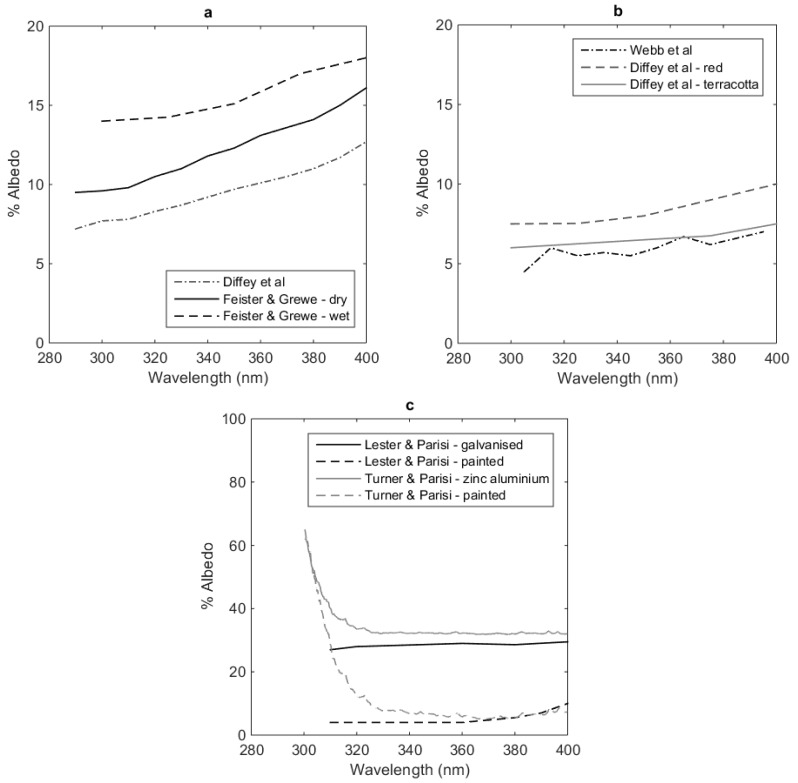
Spectral UV albedo of (**a**) concrete, (**b**) brick ground cover and (**c**) metal (steel) horizontal surfaces. The data has been collated from Feister and Grewe (F&G) [27], Diffey et al. [26], Webb et al. [36], Lester and Parisi (L&P) [30] and Turner and Parisi (T&P) [48,51].

Some of the studies included in Table 2 obtained data spectrally before reporting it as a broadband measure, while some carried out broadband and spectral UV albedo measurements separately. Studies that have investigated spectral UV albedo include [25,26,27,30,31,32,36,37,44,45,46,47]. A range of this spectral data is presented in Figure 1 (a: sand, b: earth, c: grass or vegetation, d: snow) and 2 (a: concrete, b: brick, c: metal). The metal referred to in Figure 2c is steel, with the different coatings stipulated in the figure. It should be noted here that the data reported from Doda and Green [44,45] also includes some air-based measurements as well as ground measurements. Unless otherwise specified measurements are made from ground level. Despite the focus of this review on localised measurements, it was determined that it may be helpful to include some of the airborne measurements for comparison. It is interesting to compare Webb et al. [36] and Doda and Green [44,45]. Both conducted air borne measurements (only Webb’s ground based measurements are reported here), but the variation across the spectrum changes more consistently in Webb et al.’s data compared to Doda and Green’s, presumably due to difference in technology across the changing decades. Webb et al., shows significant difference between the ground based and air borne measurements, which surprisingly in some cases, shows an increase in albedo with height of measurement, rather than a decrease. Webb et al., states that this shows that the effective albedo increases with the upwelling radiation by backscattering from the boundary layer of the atmosphere and supersedes the reflectance of the underlying surface. This enforces the argument that it is not necessarily useful to assume that upper atmosphere albedo measurements are the same as localised ground measurements.

Coulson and Reynolds [47] also performed spectral reflectance measurements on earth and vegetative surfaces, however they are not provided in Figure 1 and Figure 2. This is due to the short axis devoted to UV wavelengths in their published figures, as their study focused mainly on visible wavelengths. Values of reflectance reported in their study included loam with reflectance ranging from 5% to 10% across the UV spectrum for wet and dry loam, 8.5% to 10% for asphalt (also called blacktop) and 2% to 5% for alfalfa. Interesting features to note in Figure 1 and Figure 2 is the range of spectral measurements achieved for each surface type, and significantly, the different values reported. Different types of sand in Figure 1a show a significant range. Here Doda and Green [44,45] report ground based measurements, and are some of the highest albedo measurements in the figure. Across all their measurements is an undulating variation across the wavelengths, whereas most of the other reported spectral studies in these figures show a consistent increasing albedo with increasing wavelength. The exception to this is that reported by Turner and Parisi [48] for horizontal metal surfaces. These methods do use sun-normal UV irradiance as reference rather than downwelling UV irradiance, but it is not expected to be the reason for the discrepancy. In Figure 2c, this shows a sharp increase with decreasing wavelength for both zinc aluminium coated and painted coated steel surfaces. There are two reasons why it is believed this is demonstrated. Firstly, the data reported here is averaged from many measurements per wavelength, and secondly, the publication reports that issues with signal to noise ratios occur below 300 nm with their reported instrumentation. It is possible this issue goes above 300 nm as well. However, it is also apparent that from approximately 330 nm and up, their measurements agree with Lester and Parisi [30] whom conducted their study with a spectroradiometer.

From Figure 1 and Figure 2, it appears that the surfaces with greatest range in magnitude in spectral UV albedo measurements, are sand and snow, although grass shows some variation as well. The reason for this could also be as simple as that there are not enough measurements from other surface types to show variation in measurement. Warren [38] discusses the optical properties of snow although focuses more across the solar spectrum rather than just the UV spectrum. In general, for visible radiation, the albedo decreases as wavelength increases, however when looking closely at the UV spectral data in the report (which is small in relation to the visible spectral reported data), readers of that report can see that in the UV spectrum, UV albedo increases for increasing wavelength which correlates to the data reviewed here. Meinander et al. [49] looked at the diurnal variation in snow albedo in Finland, including grain size, but as they investigated this using broadband meters, were unable to determine if change in albedo due to grain size was due to the change in spectral nature or some other factor.

Another study not included in Figure 1 and Figure 2, is Brandt et al. [50] who measured surface albedo of Antarctic Sea ice through a variety of measurement techniques, including thick ice measured at 1 m from a suspended rod over the side of a ship, or by measurements from a hovering helicopter up to 25 m from the ice or sea surface. Unfortunately, it is not possible to tell from the study which measurements correlate to which data collection method. Most data collected was reported to occur under sun obscured skies, therefore the albedo measured is diffuse. The data reported shows the spectral UV albedo, is a minimum of 70% for thick first year ice (140 cm), to 7% for thin 2.2 cm ice. Open water was reported as being independent of wavelength and averaging also at 7% across the UV spectrum. Snow cover on ice, increased from 70% with no snow, up to 90%, which agrees with many of the above reported studies.

### 3.3. UV Reflectance

Historically, there has been significant interest in UV reflectance from man-made structures, although the research based in the early 20th century was more interested in daylighting and germicidal benefits, with metals considered to increase solar exposure for health related reasons. Many different metal types were investigated spectrally. Some of the earliest measurements are reported in 1915 in The Astrophysical Journal [52] and continue up until the Second World War [53,54,55,56,57,58,59,60] and also cover pigment and paint reflectance as well as metal reflectance. In many of these studies, UV wavelength ranges extended into the UVC (<280 nm), in order to specifically determine the usefulness of these surfaces to reflect germicidal UV irradiance. Luckiesh [61] reports in a chapter on reflection in ultraviolet radiation that the following metals or elements were investigated and characterised for UV reflectance by Hulburt [52]: aluminium, antimony, bismuth, cadmium, carbon, carborundum, chromium, cobalt, copper, gold, lead, magnalium (69:31 aluminium to magnesium), magnesium, molybdenum, nickel, palladium, silicon, silver, speculum (68:32 copper to tin), steel, stellite, tantalum, tellurium, tin, tungsten and zinc. Luckiesh also summarises many other studies of his time in this chapter.

Table 5 takes more commercial interests in UV reflectance, and seeks to combine similar surface types for comparison. The alignment of colour names in Table 5 was achieved by using the description of the colour as well as the solar, visible (VIS) and near infrared (NIR) reflectance to guide alignment of the differently named coloured asphalt tiles. This review assumes that each study used a similar radiant source, which does not appear to be reported, although each report the use of the same method using a spectrophotometer (single pass monochromators with diffraction grating) with integrating sphere, although not the same model of equipment. Each study adheres to using the methodology recommended by the Active Standard Test Method (ASTM) E903. The work by Berdahl and Bretz [22] and Parker et al. [33] are measurements of reflectance rather than albedo. Parker et al., has conducted more reflectance measurements over more colour coated types. This study additionally carried out tests over multiple types of surface and some of these UV reflectance measurements are presented in Table 4, including new unpainted aluminium (75%), unpainted galvanised tin (29.3%), smooth bitumen (4.2%) and granular surface bitumen (9.3%). This study is particularly interesting in that both the terms asphalt and bitumen are used separately for roofing materials. Therefore this suggests there are differences in the composition of these types of materials. One other interesting measurement is the measurement of the thermal reflective coating product Insultec (Insultec Australia) on a metal swatch, measured at 18.4% UV reflectance. Turner [48] made measurements on a similar vertical surface but only measured spectral UV reflectance. Values measured by Turner ranged above and below the Parker et al., measurements.

Previous work by the authors Turner and Parisi [20,46,51] have looked at the measurement of UV reflectance from building materials. The early work initially focused on reflectance from vertical surfaces, in an attempt to measure albedo [46], however it was eventually determined that the method of albedo measurement resulted in artificially inflated values [20] and needed to be adjusted to compare reflectance from differently oriented surfaces. The adjustments required for a non-horizontal surface was simply the need to use a sun normal measurement of irradiance as a reference, and a surface normal measurement to derive a ratio with respect to the reference measurement. This accounts for direction of incident irradiance. A comparison between horizontal and non-horizontal surface reflectance was then successfully made [51] with some basic relationships calculated between the surface type. These measurements and calculations were limited to metallic surfaces, which is a common building material in Australia. The research demonstrated that the zinc aluminium coated steel reflected strongly in the UV spectrum. Paint coated steel had a much reduced UV reflectance in comparison since paint coated metal surfaces have lower influence on exposure compared to non-coated metal surfaces.

## 4. Measurement Design

Table 2 also provides the range of measurement design carried out within the UV albedo studies. Most measurements were made with a broadband device. Some studies conducted sequential measurements with a single sensor, while others combined two sensors oriented to capture upwelling and down-welling irradiance at the same time of measurement. Distance from the surface seemed to be the most variable amongst the reported studies which is surprising. The inverse square law of light tells us that the intensity of light decreases with increasing distance. However, Webb et al. [36] shows that increased distance from a surface may reveal increased reflectance, not due to the surface itself, but due to the intervening atmosphere which itself decreases or increases radiative transfer. UV radiation is more likely to scatter compared to longer wavelengths due to the Rayleigh criterion, thus decreasing directional UV radiation. Except for surface albedo measurements made from higher up in the atmosphere, UV albedo should not be dependent on distance from surface measurement, however this would not be the case for UV reflectance. In the case of strongly directional reflectance surfaces, direct UV reflectance would decrease with increasing distance from a surface.

Consistency amongst method of albedo measurements therefore appears to be less crucial when compared to measurements that show changing albedo for a single surface, or reflectance measurements. However, the work by Weihs [16] might suggest that in retrospect, all albedo measurements for surfaces that are known to vary due to apparent specular reflectance from a surface, should be made over varying solar zenith angle (SZA) in order to confirm the surface is producing UV albedo rather than UV reflectance. This will be particularly relevant to researchers who rely on UV albedo to be correct for modelling scenarios. However, it appears that for some modelling scenarios, a value is not required. A recent paper that investigates influence of reflective surfaces is [62] that models the UV environment at the ocean. The study suggests that reflectance from the sea surface accounts for less than 20% of the UV exposure on a vertical surface. What is interesting is that a comparison against an actual measured albedo was not conducted, however, given the complexity in the scenario the model seeks to describe, it is understandable that empirical evaluations are not attempted. The model found the contribution of the sea surface reflected irradiance ranged from less than 13% (for hazy sky) to less than 16% (for clear sky). Given that albedo in this review (Table 3) shows sea surface reflectance ranging from 7% [50] to 30% [14,29] it would be interesting to know how the albedo effectively contributes to overall exposure.

## 5. Influence on Personal UV Exposure by Reflected UV Radiation

There are many studies that have investigated the factors that influence UV radiation exposure, ranging from all the many composite variables, such as clouds, SZA and ozone. However specific studies that seek to determine definite changes to personal UV exposure due to albedo from surfaces are limited. There are a range of studies that specifically mention UV reflective surfaces but do not seek to specifically measure that component derived due to UV albedo exactly [63,64,65,66,67]. One could argue that it would not be necessary in these complex environments, as more than just albedo will have significance. These studies range from occupational exposure [63,65], exposure obtained on a beach under a beach umbrella [64] and exposure obtained in an alpine site [67] and scientists in an arctic site [68]. While no albedo was measured in this latter study, the research concluded that scientists in the field received twice the UV exposure compared to scientists mostly working in tents in the field. McKenzie et al. [69] through modelling found that albedo of snow at approximately 80% resulted in an enhancement of 40% UV exposure. Kylling et al. [70] through model simulations concluded that monthly erythemal doses should increase by more than 20% due to surface snow in high latitude sites. Daily dose changes due to albedo from snow, were variable but overall the study concluded that over a year, albedo in this snow prone site could increase exposure by 8%. Some studies look at the change in irradiance due to effective albedo. Effective albedo is defined as the ratio of the reflectivity of a surface compared to a low reflective surface (such as less than 20% albedo) and is mostly used in snow prone sites. Comparing albedo to effective albedo can add an additional complexity between studies, and the reporting of effective albedo can then depend on peak wavelength for effective albedo. Such wavelengths might be 305 nm resulting in a 57% increase in downwelling irradiance [71], 320 nm resulting in a 15% increase in global irradiance relative to a coastal site [72] and 340 nm resulting in a 15% increase in average irradiance compared to a coastal site [73]. To compare this information successfully we have to refer to modelled albedo [74,75] which “for a uniform Lambertian surface the amplification factor for the global irradiance depends only on the product of the surface reflectance and the atmospheric backscatter. It varies with wavelength, reaching a maximum near 320 nm; this maximum is close to 50% for clean snow” [74]. While these show impact on global irradiance, downwelling irradiance or average irradiance, the studies do not provide any further information about exposure rates without additional complex analyses.

Specific studies that seek to understand the exposure to occupational workers from albedo influence are limited. Weber et al. [76] looked at the average exposures of tinsmiths, who regularly work outside under full sun, above highly UV reflective surfaces. The data obtained did not seek to measure the known albedo, rather focused on the overall exposures of the workers, and in turn seek to educate the workers over personal protective measures [76]. Turner and Parisi [51,77] looked at how much influence a reflective vertical metal surface (zinc aluminium coated steel and paint coated steel) could affect the personal exposure of construction workers, using dosimetry and manikins. They concluded that overall, at certain times of the day, total UV exposure could be increased by 20%, while facial positions may incur a 50% increase in UV exposure due to directional irradiance. A corner structure (two façades with a 90º vertex) as opposed to a single façade did not appear to increase UV exposure further [78]. Whilst reflectance measurements were taken at the same time as UV exposure measurements, the authors did not correlate reflectance measured with influence to UV exposure. Liu et al. [79] looked at the enhanced ocular UV irradiance from a beach surface as compared to a grass surface. They measured twice the biologically effective irradiance on a beach surface compared to the grass surface, occurring at a maximum of a solar zenith angle of 40º. Lester and Parisi [30] found that zinc aluminium coated steel surfaces produced enhancements magnitudes larger than that of the paint coated steel or grass surfaces. Manikin head forms were placed vertically over horizontal surfaces with attached dosimeters. Sites such as under the chin experience significant increases. The enhancement was reported as 1286% times larger compared to grass, whilst the nose and cheeks were enhanced by 190% and 140% respectively. This study also measured the albedo of the surfaces spectrally but did not appear to link the albedo to the enhanced UV exposure through any model. Parisi et al. [80] modelled the enhancements due to UV albedo with changing SZA, from measurements made with a rotating vertical positioned manikin. They found that for three different surface types (water, concrete and sand), inclined at planes ranging from 30° to the horizontal reflective surface, to −45°, a range of enhancements were possible. Maximum enhancement was observed at −45° inclined surfaces. Water ranged from 0.6% to 5.2%, concrete ranged from 1.1% to 9.4% and sand ranged from 1.8% to 15%. It is interesting to note the enhancements found at 90° to the horizontal, with 3.7% for water, 6.8% for concrete and 10.9% for sand. These are useful when considering the orientation of surfaces of a standing person.

It is apparent then that whilst the study of influence of albedo contribution to UV exposure is relevant, there is no study that has sought to correlate albedo or reflectance values with the actual increased UV exposure. From the literature we can see that high albedo from snow sites do not necessarily result in a directly proportionally higher exposure to the albedo value. This may be due to the surface being horizontal, or inclined, and the receiving surface vertical (in the example of skiers) [67]. For vertical metal surfaces which have a high UV reflectance, it does not appear that UV reflectance immediately correlated with a proportionally high UV exposure [77]. In these two latterly mentioned studies, dosimeters were used to determine exposure change, compared to simulation models. This leads to questions such as, how does the physical value of UV albedo or UV reflectance, contribute to the change in UV exposure to an individual? Can it be quantified and will it change the way simulated models should be used in calculating changes to individual UV exposure? These questions suggest that UV albedo and UV reflectance should continue to be empirically measured, alongside changes in UV exposure due to albedo or reflectance, to better resolve the interaction between these aspects. It may also assist in improving the use of UV albedo and UV reflectance in simulation models to determine UV exposure.

## 6. Conclusions

The definitions of albedo and reflectance have been used interchangeably in the past, however this review suggests that recognising the conditions and assumptions surrounding the definition of UV albedo, helps distinguish between it and UV reflectance measurement. Even then, it can be difficult to use the terms consistently, especially given the range of the way the terms are used in the literature. In general, this review recommends that albedo be used for measurement of natural mostly horizontal surfaces, with the condition that the albedo does not change significantly during the day. Reflectance measurements should be used for non-natural surfaces and non-horizontal surface measurements and will change according to the varying conditions including the geometry of incoming and reflected irradiance, as well as due to the intrinsic nature of the surface itself. Urban studies may benefit most from this use. The review of the existing literature on UV albedo shows that only a handful of surfaces have been measured more than a few times and published, whereas many different surface types have been measured only once for UV albedo. This applies for both broadband and spectral UV albedo measurements. Comparison of the spectral data across publications have been presented graphically where possible. Even in a single report, conflicting UV albedo measurements can be presented, therefore the compilation of all known data collected and published here, aims to present all known information available to date. The method of measurement is much more crucial for reflectance measurements, and needs to be clearly stipulated in order to produce repeatable results. Albedo, assuming it adheres to the assumptions and conditions surrounding its definition, can be measured fairly consistently despite differences in distance between surface and sensor amongst methods used. However, it is still recommended that albedo measurements be accompanied by a detailed method of measurement. A review of studies that seek to understand high albedo sites that influence UV exposure was conducted, however there does not appear to be studies that have sought to correlate albedo or reflectance with actual influence to UV exposure. This presents an opportunity for researchers to continue exploring the influence of reflective UV surfaces.

## Figures and Tables

**Table 1:** Definitions of descriptors of reflectance as provided by Hapke [11].

Term	Behaviour of Radiation from Source or Recorded at Detector
Directional	Highly collimated (narrow focus beam)
Conical	Medium collimated source (may be considered broad beam)
Hemispherical	Limited collimated source

**Table 2:** Publications with reportable localised UV albedo measurements including methodology. The identification number (ID) is used to identify the measurements compiled in Table 3 and Table 4 per study.

ID	Author-Date Identifier	Instrument Used	Light Source	Measurement Type	Method	Location	Distance from Surface
1	Diffey et al., 1995 [26]	Double GaP photodiode Radiometer Calibrated to spectroradiometer + diffuse reflectance spectrometer	Sun	Broadband and spectral (relative to standard white reflectance surface)	Upwelling and downwelling irradiance measured concurrently	Canada England Saudi Arabia	Not stated
2	Reuder et al., 2007 [34]	Radiometer (UV-S-E-T 001) Erythemal radiation reflected	Sun	Broadband	Increase in UVIndex (ratio)	Salar de Uyuni, Bolivia	2.0 m from ground
3	McKenzie 1996 [32]	Erythemal monitor spectroradiometer	Sun	Broadband	Upwelling and downwelling irradiance measured consecutively	New Zealand	Assumed 1.6m height comparable to spectral Grass approx. 30 cm height
4	Blumthaler & Ambach 1988 [23]	R-B meter, Star pyranometer	Direct sunlight & overcast sky	Broadband	Erythemal albedo. Also: Total solar (0.3 to 3 µm) albedo consecutive	Alpine regions Austria	0.3–0.5 m from ground
5	Lester & Parisi 2002 [30]	Spectro-radiometer (integrated)	Sun	Spectral & Broadband + Biologically weighted	Upwelling and downwelling, sun normal, consecutive	Toowoomba, Australia	1.7 m from surface
6	Feister & Grewe 1995 [27]	Spectro-radiometer (integrated up to 315 nm) OL752/10 Optronic Laboratories Biologically weighted for different spectra	Sun	Spectral	UVB Upwelling and downwelling irradiance measured consecutively	Germany	2.0 m from ground
7	ICNIRP 2007 [29] a—reported in text Section 2.2.2 b—reported in Section 4.4 and Table B-7	Unknown Diffuse reflectance ICNIRP effective solar UVB	Assumed sun	Broadband	Not stated	Not stated	Not stated
8	Sliney 1986 [14]	International Light Model 730 UV radiometer (295–315 nm) calibrated to spectroradiometer	Sun	Broadband	Upwelling and downwelling irradiance (diffuse) measured consecutively	Not stated	Not stated
9	Heisler & Grant 2000 [28]	Refer to ID 4 [23] & ID 8 [14]					Reports Blumthaler and Ambach 1998 [23] and Sliney 1986 [14]
10	Rosenthal 1988 [35]	SCS280 detector Radiometer (295 nm to 350 nm)	Assumed sun	Broadband	Upwelling and downwelling measured consecutively		Upwelling measure 4 feet from ground
11	Correa & Ceballos 2008 [25]	UVB Biometers (501) × 2 1 inverted—“albedometer”	Sun	Broadband	Upwelling and downwelling irradiance measured concurrently	Brazil	Upwelling measure 0.4 m from ground
12	Webb et al., 2000 [42]	Spectroradiometer—Bifurcated optics (top and bottom of Cessna)	Sun	Spectral	Upwelling and downwelling irradiance measured concurrently	North-west coast England	Upwelling irradiance (0.5m from ground) Downwelling irradiance (1.1 m from ground) Air based ranging from 700 ft to 5500 ft
13	Castro et al., 2001 [24]	Eppley radiometer (Model 8-48)	Sun	Broadband	Downwelling to upwelling	Mexico City urban and rural	Height not stated
14	Lin et al., 2011 [31]	(B&W Tek, BRC112F) spectrometer	UVB lamps Philips TL20W/01	Spectral converted to broadband	Reflectance reference from silicon standard	Laboratory	0.1 m from surface
15	Parker et al., 2000 [33]	Beckman 5240 Spectrophotometer with integrating sphere	Solar exposure	Broadband	Reflected irradiance to incident irradiance (15°)	Florida, FL, USA	Not stated

**Table 3:** Broadband UV albedo measurements of mostly natural surfaces in percentages as reported in the literature (Table 2). ID numbers in the top row correspond to the ID numbers assigned in Table 2.

% Albedo ID	1	2	3	4 & 9	5	6	7a	7b	8 & 9	10	11	12	13	Min	Max	Mean
Surface Type																
Loam						4.4								-	-	4.4
Bare ground			3.2						4–6 ^a^				3.9	3.2	6	4.3
Salt lake		69 ± 2												-	-	69
Sandy soil	5.9												2–3	2	5.9	3.63
White sandy soil	9.1															9.1
Sand (freshwater)	8.9			9.1		15.2	15–30 ^b^		7.1 ^c^					7.1	30	14.22
Beach sand (wet)								7			2.4 ^d^			2.4	7	4.7
Beach sand (dry)								15–18	15–18		4.2 ^e^			4.2	18	14.04
White clay													12	-	-	12
Primitive rock				3.7										-	-	3.7
Limestone				11.2										-	-	11.2
Flower bed	2.6													-	-	2.6
Mown grass	1.8 ^f^ 1.2 ^g^ 1.4 ^h^		0.8–1.2					2.0–3.7			1.1 ^i^ 1.0 ^j^	0–1.6	1–5	0	3.7	1.75
Long grass			0.5–1.0	1.3		1.7								0.5	1.7	1.07
Lawn					1–3	2.4			3.7	1.1–1.4				1	3.7	2.1
Alfalfa													1.8	-	-	1.8
Clover			0.8											-	-	0.8
Pasture				4.9				0.8–1.6						0.8	4.9	2.43
Oats						1.7								-	-	1.7
Rye						1.7								-	-	1.7
Straw													4.3	-	-	4.3
Wheat													1	-	-	1
Lake side water	3.2			4.8						2.7–3.9				2.7	4.8	3.65
River side water	3													-	-	3
Fresh water over gravel (0.5 m)			1.8											-	-	1.8
Surf							20	25–30	25–30					20	30	26
Snow						76.2	90	88	88					76.2	90	85.55
Dirty snow								59						-	-	59
New dry snow				94.4					85					85	94.4	89.7
New wet snow				79.2										-	-	79.2
Old dry snow				82.2					50					50	82.2	66.1
Old wet snow				74.4										-	-	74.4
Most ground surfaces							>10							-	-	

^a^ clay, ^b^ Gypsum sand, ^c^ beach, ^d^ wet coarse sand, ^e^ dry coarse sand, ^f^ Canada, ^g^ England, ^h^ Saudi Arabia, ^i^ green grass, ^j^ yellow grass.

**Table 4:** Broadband UV albedo measurements of mostly man-made surfaces in percentages as reported in the literature (Table 2). ID numbers in the top row correspond to the ID numbers assigned in Table 2.

% Albedo ID	1	3	4 & 9	5	6	7b	8 & 9	10	11	12	13	14	15	min	max	mean
Surface Type																
Concrete (new)		15.8			9.8		10–12	14.6						9.8	15.2	12.44
Concrete	8.2	9.2				8–12	7.0–8.2				10–11 ^k^		9.7	7	12	9.26
Wet concrete					8									-	-	8
Concrete/pebble tile	12.4													-	-	12.4
White concrete tile													22	-	-	22
Ceramic tile—porcelain —stoneware —vitrified mosaic												11.48 17.30 33.22		- - -	- - -	11.48 17.30 33.22
Gravel path	8.2	5.8												5.8	8.2	7
Asphalt			5.5			5–9 black	4.1–5.0 ^l^ 5.0–8.9 ^m^				2-7		4.2–9.2	2	9	5.90
Tar sealed road		6												-	-	6
Tarmac road	6.5 ^f^ 5.5 ^g^ 5.7 ^h^									9.8–15 ^n^ 9–9.8 ^o^				5.5	15	8.76
Tennis court			2.9											-	-	2.9
Wooden boards (dock)	4.4					5-7	6.4							4.4	7	5.7
Natural clear wood									2.6				5.2	2.6	5.2	3.9
White painted wood									4.2					-	-	4.2
Black painted wood									2.7				6.5	2.7	6.5	24.6
Enamel paint (white/red)		5.1												-	-	5.1
Black butyl rubber roof		5.1												-	-	5.1
Stainless steel opaque plate									4.3					-	-	4.3
Steel plate—colour coating												8.86 ^s^–13.35 ^t^		8.86	13.35	11.11
Shiny corrugated iron		18.1		30 ^p^ (25– 32) ^q^										18.1	30	24.05
Pale pink corrugated iron				3–12 ^p^ (3–6) ^q^										-	-	7.5
White paint—metal oxide							22						17.5	17.5	22	19.75
Aluminium-weathered							13						75 ^r^	13	75	44
Unpainted galvanized tin													29.3	-	-	29.3
White fibre glass							9.1							-	-	9.1
White formica									7.9					-	-	7.9
Polycarbonate hollow sheet												8.46		-	-	8.46
Pottery wall tile												12.35		-	-	12.35
Red brick										4.5–7				4.5	7	5.75

^f^ Canada, ^g^ England, ^h^ Saudi Arabia, ^k^ cement, ^l^ old, ^m^ new, ^n^ light tarmac, ^o^ dark tarmac, ^p^ broadband, ^q^ biologically weighted, ^r^ new not weathered, ^s^ cream coloured fire resistant coating, ^t^ grey-composition not stated.

**Table 5:** Comparison of reflectance measurement as a percentage in different broadband wavebands, for similar commercial products by [22,33]. The divisions are reported by Parker et al. [33] as total solar reflectance (300 nm to 2500 nm), UV reflectance (300 nm to 400 nm), visible (VIS) reflectance (410 nm to 722 nm) and Near Infrared (NIR) reflectance (724 nm to 2500 nm). Divisions not reported by Berdahl and Bretz [22].

Berdahl & Bretz	Solar	UV	VIS	NIR	Parker et al.	Solar	UV	VIS	NIR
Asphalt Shingle Reflectance					Shingle Colour				
black	5	4	5	5	Generic Black	5	4.6	5.3	4.8
					Onyx Black	3.4	3.7	3.5	3.3
white	21	6	24	21	Generic White	25.3	9.9	27	25.2
					Shasta White	26.1	11.5	29.6	24.2
					ISP K-711 “white”	31.1	12.2	34.4	29.9
					Generic Grey	21.7	10.1	23.1	21.7
gray	8	6	8	9	Ocean Gray	11.7	7.2	12.3	11.5
antique silver	20	6	22	19	Aspen Gray	17.8	8.9	19.5	17.2
saddle tan	16	5	16	18	Desert Tan	12	4.3	11.3	13.5
light brown	19	7	19	20	Beachwood Sand	20	7.5	20.5	20.8
medium light brown	10	5	10	11	Island Brown	8.7	4.4	7.8	10
medium brown	12	6	12	12	Autumn Brown	9.6	3.9	8.6	11.1
dark brown	8	5	8	9	Weathered Wood	8.2	5.4	8.4	8.3
green	19	8	21	20	Surf Green	15.7	9.1	16.2	16.1
Commercial roof coatings									
Koolseal elastomeric	81	14	88	81	Kool Seal Elastomeric over shingle	71.4	16.7	80	69.1
MCI elastomeric	80	12	87	81	Aged Elastomeric on plywood	72.7	17.4	78.5	73.1
					Flex-tec Elastomeric on shingle	65	14.1	69.4	66.3
